# Epidemiology and Characterization of Shiga Toxin-Producing Escherichia Coli of Hemolytic Uremic Syndrome in Argentina

**DOI:** 10.7759/cureus.17213

**Published:** 2021-08-16

**Authors:** Jeronimo F Torti, Paula Cuervo, Andrea Nardello, Marcela Pizarro

**Affiliations:** 1 Biochemistry, National University of Cuyo, Mendoza, ARG

**Keywords:** shiga toxin-producing escherichia coli, hemolytic uremic syndrome, argentina, molecular pathogenesis, emerging pathogen

## Abstract

Argentina has one of the highest prevalence in Shiga toxin-producing *Escherichia coli* (STEC) and the high rate of hemolytic uremic syndrome (HUS) in the world. Though preventive steps such as food safety have been implemented as a way to reduce STEC infections, these have proven to be insufficient. STEC's pathogenesis, virulence factors, relationship with the environment, and emerging strains have been studied in the past few years in the country. Many factors that contribute to the morbidity and mortality of STEC infections include the expression of pathologic genes, alternative characteristics (inhibition of phagocytosis, invasion, cytotoxicity, and bacterial attachment), and host factors (age, immune status, treatments, medical history). However, research studies in combination with epidemiological data suggest trends of the prognosis, with the relationship between and genetic combinations of adherence, Shiga toxin (Stx) genes, and virulence genes, which significantly influence disease outcomes. This review explains the characteristics and epidemiology of STEC in Argentina. All these facts show that the application of molecular subtyping techniques in real-time is essential for detecting and controlling outbreaks. Applying molecular subtyping techniques in hemorrhagic diarrhea can avoid severe consequences caused by progression to HUS, and help the epidemiological analysis of the outbreak.

## Introduction and background

*Escherichia coli *(*E. coli*) is part of the healthy microbiota of any mammal gastrointestinal tract. However, some *E. coli* strains acquire new genetic material, and they become pathogenic. Pathogenic *E. coli* is one of the major causes of infections in humans' gastrointestinal tract, where it can cause different types of diarrhea. Also, it can span to extraintestinal sites. Extraintestinal pathogenic *E. coli* (ExPEC) can cause diseases such as septicemia, urinary tract infection (UTI), central nervous system infections such as meningitis in newborns, and respiratory system infections [1]. Intestinal *E. coli* (InPEC) pathotypes are enteropathogenic *E. coli* (EPEC), enteroinvasive *E. coli* (EIEC), enterotoxigenic *E. coli* (ETEC), enteroaggregative *E. coli* (EAEC), diffuse adherent *E. coli* (DAEC), Shiga toxin-producing *E. coli* (STEC), and adherent-invasive* E. coli* (AIEC) [1].

Shiga toxin-producing *E. coli *(STEC; also known as verotoxin-producing *E. coli* {VTEC}) is a group of *E. coli *that express the genes of Shiga toxin (Stx) [2].​​ These genes are typically acquired by a lambdoid bacteriophage [2]. STEC is the most critical recently emerged group of foodborne pathogens. STEC can produce severe human illnesses, such as diarrhea, hemorrhagic/bloody diarrhea (BD), and hemolytic uremic syndrome (HUS). Furthermore, 5% to 15% of BD progress to life-threatening HUS associated with ischemic organ damage [1]. 

Stx came to medical attention in 1983 with two nearly simultaneous reports, one of which identified *E. coli* O157:H7 in the stools of patients with BD, who had been exposed to undercooked hamburgers, where they found a toxin from the *E. coli* O157:H7 strain, that was structurally similar to the toxin of *Shigella* *dysenteriae* type 1, and named it Shiga toxin [3]. The other study identified *E. coli* O157:H7 and a toxin-producing *E. coli* (STEC) belonging to other serotypes in the stools of children with hemolytic uremic syndrome (HUS). This toxin destroyed Vero cells (epithelial cells isolated from the kidney of an African green monkey), therefore they name verotoxin [4]. Both terms still apply to describe the same toxin.

Microbiology

*E. coli* strains and lineages are classified by their "O" and "H" antigens [5]. The O (ohne) antigen is part of the lipopolysaccharide (LPS) embedded in the outer leaflet of the bacteria's outer membrane and is defined serologically and determined by the repeating polysaccharide chains. The H (hauch) antigen is defined serologically by the antigenic specificity of the bacterial flagellum. The O and H antigens are used most frequently in the serotyping of pathogenic *E. coli* because they have the best correlation with virulence factors [5]. Members of a clone of bacteria that express the same O antigen are described as a serogroup. When the serogroup and the H (flagellar) antigen are mentioned in unison, the designation becomes a serotype. However, there are now new typifying methods of sequencing genetic technologies. Furthermore, whole-genome sequencing (WGS) of *E. coli* is establishing subtypifying methods now. 

*E. coli *O157:H7 is the most commonly isolated STEC serotype worldwide. This serotype's predominance partly relates to the ease with which this serotype can be detected on sorbitol MacConkey (with or without supplementation with cefixime and tellurite) and does not ferment sorbitol when grown on agar plates containing sorbitol as a carbon source. This easily detectable property facilitated an early understanding of the epidemiology and spectrum of infections caused by these serotype strains. Several other *E. coli* serotypes that produce Shiga toxin ("non-O157:H7 STEC") have been identified less frequently because few possess a phenotype as distinctive as the inability to ferment sorbitol on agar plates. These STEC are associated with a broader spectrum of illnesses than *E. coli* O157:H7 and are usually less severe [6]. Techniques used to detect non-O157:H7 STEC serotypes include toxin enzyme immunoassays of overnight broth cultures of stools and nucleic acid amplification of Shiga toxin genes directly from the stool, broth, or isolated colonies [6]. Nevertheless, *E. coli* O157:H7 remains the leading serotype of STEC recovered worldwide, and, in general, causes more severe disease than non-O157:H7 STEC, where we have a proportion of 0.823% in HUS and 0.045% in mortality in *E. coli* O157:H7 cases versus a 0.025% in HUS and 0.002% in mortality in nonO157:H7 STEC cases [7]. 

According to a polymerase chain reaction (PCR)-based assay and polyacrylamide gel separation study, *E. coli *O157:H7 have three genetic lineages (I, II, and I/II) [8]. It is thought that this is the result of an ancestral clone and a subsequent regional expansion. Latter studies disclosed differences between the three lineages including Stx2 expression, Stx-encoding bacteriophage insertion sites, and stress resistance as well as lineage-specific polymorphisms [9]. To investigate the characteristics that predispose to a serious disease in the differents STEC O157:H7 strains, Manning et al. analyzed a collection of 519 STEC O157s, 96 single nucleotide polymorphisms (SNPs), located in 83 genes [10]. The phylogenetic analysis of these SNPs allowed the profiles to be grouped into 39 genotypes, separated into nine different clades. SNP typing not only provides stable genetic markers to study evolution but also offers a higher phylogenetic resolution. SNP discovery approaches have yielded thousands of high-quality SNPs as critical bases for developing a refined phylogenomic framework. They found that more than 75% of the studied strains that produce disease belonged to clades 2, 3, 7, and 8. Differences were observed between clades in the frequency and distribution of Shiga toxin genes and the type of clinical disease reported. Patients with the HUS were significantly more likely to be infected with clade 8 strains, which have increased in frequency over the past five years (seven times more likely to develop HUS than other clades combined) [10]. Hospitalized young patients (under 18 years) frequently possess Clade 8 strains, which are highly associated with the stx2a/stx2c genotype [10]. Clade 8 of O157:H7 has been considered the most hypervirulent clade, and it is responsible for a more severe and rapidly progressive disease, which would make diagnosis difficult during the diarrhea phase. Patients with the HUS were significantly more likely to be infected with clade 8 strains. The correlation between clade typing and genetic linage is that lineage II correlates to clade 7, lineage I/II correlates to clade 8, and lineage I correlate to clades 6 through 1 [11]. In addition, others studies showed differents virulence factors expressions between different clades, and WGS studies elucidated further potential virulence determinants [11]. WGS of the spinach outbreak in the United States in 2006 (strain TW14359), a member of clade 8, also revealed substantial genomic differences. These findings suggest that a new subpopulation of the clade 8 lineage has acquired critical factors contributing to more severe disease [12]. They found seven coding sequences postulated as “putative virulence factors” that increase this strain´s virulence [12]​​​​​​. 

The most common non-O157 serogroups in Europe comprehend O145, O146, O91, O103, and O26 and in the United States, there are O121, O45, O26, O111, O103, and O145. In 2011, O104 caused an outbreak in Europe, which was an EAEC strain that had acquired the Stx2 encoding bacteriophage [13]​​​​​​. Moreover, four EAEC-STEC strains nonidentical serotypes were reported, between 1992 and 2012, that create six sporadic cases of HUS and one outbreak [6].

Pathogenesis

The defining factor of STEC is its ability to produce extracellular Shiga toxins (Stx) known as Shiga toxin 1 (STx1) and Shiga toxin 2 (STx2) [14]. Although, the Shigella species is a separated genus, there are phylogenetically within the clade *E. coli* [14]. STx is an AB5 toxin structure that inhibits protein synthesis in sensitive eukaryotic cells. The pentameric B subunit binds a sphingolipid, globotriaosylceramide (GB3), on eukaryotic cell surfaces, and an enzymatically active A subunit that enters the cell. Stx is released from STEC in the intestine, most probably during bacterial lysis [15]. Prophages that are integrated into the chromosome encode either Stx1 or Stx2. Stx1/Stx2 is released from lysed bacterial cells during the lytic cycle of the phage during stress [15]. Clinically, the use of antibiotics is not recommended due to the lytic cycle's stimulation that can produce and concomitant toxin release. Investigations have demonstrated that fluoroquinolones improve Stx2 production in STEC O157:H7. Furthermore, there is evidence that subinhibitory concentrations of trimethoprim and fluoroquinolones produce the lytic cycle, while other antibiotics such as azithromycin did not [16].

Stx1 and Stx2 each have multiple allelic genotype variants with different associations with human disease. The subtypes are classified according to amino acid sequences of the Stx. Stx1 has four subtypes - *Stx1a, Stx1c, Stx1d, and Stx1e* [6]. Stx2 has 12 subtypes - *Stx2a *to *Stx2*l [6]. Both Stx1 and Stx2 can cause HUS; however, Stx2 is frequently related to severe illness [17]. Into the bargain, Stx2a has shown the highest rates of HUS, hospitalization, and BD, but, all Stx subtypes were related to some kind of severe disease. Therefore, all STEC can be pathogenic [6]. Differences in the capacity to regulate cytokine/chemokine expression during the innate immune response, as well as differences in the ability to induce cell death, may explain the greater potential of *Stx2a* to cause extraintestinal complications such as BD with HUS and central nervous system abnormalities, and fatality in humans [17]. Besides, a strain that portate the Stx gene does not mean that it would express it. It is very complex the pathogenesis of the STEC because requires the expression of a variety of genes, even genes that are not directly associated with virulence. In addition, the environment impacts the expression of virulence genes. [17].

In 1998, Nataro and Kaper proposed that severe disease was associated with STEC types that produce attaching and effacing (AE) lesions on the intestinal mucosa, and a with 60-MDa plasmid [14]. Historically, recognized as enterohemorrhagic *E. coli* (EHEC). However, the EHEC terminology is now obsolete. The 60-MDa plasmid commonly found in O157:H7 strains contains genes (*ehxA*) encoding a hemolysin (termed enterohemolysin) [14], but is widely distributed among nonO157 STEC [17].​​​The locus of enterocyte effacement (LEE) pathogenicity island has all the genes of the proteins involved in the AE lesion [18]. The LEE encodes for intimin (eae gene), a type III secretion system (EspA, EspB, and EspD), translocated intimin receptor (Tir), and effector proteins translocated by the secretion system. The eae gene is used as a surrogate marker for the LEE region. AE-lesion formation is crucial for the pathogenesis of LEE-positive STEC but it is not primordial to produce severe disease, since LEE-negative STEC strains such as O113:H21, O91:H21, and O104:H21 have been found from patients with BD and HUS. All STEC strains can be pathogenic, causing at least diarrhea, and all STEC subtypes may be associated with severe illness, i.e., HUS, BD, and/or hospitalizations [6]. As more LEE-negative are described, proteins such as the autoagglutinating adhesin (Saa), which are expressed by O91:H21 and O113:H21, have been exposed as colonization factors [19]. Furthermore, 13 ExPEC-STEC strains (cross-pathotype) of cases of UTI and diarrhea were found with *Saa* [6]. Otherwise,* aggR *encoding a bacterial transcriptional regulator, a typical virulent factor for pathogenic EAEC, has been founded in a subset of LEE-negative STEC [6]. Studies with WGS in strains non-LEE have identified several pathogenicity islands (PAIs). These genes have a variety of functions including invasion, inhibition of phagocytosis, bacterial attachment, and cytotoxicity [6].

In conclusion, there is no minimum combination of genes required to cause severe illness because there are many contributory factors, such as host factors (age, immune status, treatments, medical history), gene expression levels, and alternative genes, performing similar functions. However, the relationship between Stx genes, adherence, and other virulence genes or gene combinations can suggest general trends of disease outcome.

## Review

Epidemiology and characterization of STEC in Argentina

About one-third of *E. coli* O157:H7 infections occur in patients aged 20 years to 59 years [7]. However, the greatest burden of HUS occurs in children less than five years of age, followed by adults more than 60 years old [20]. The reasons why these age groups are most commonly and severely affected are unknown, but potential explanations include exposure, transmission, the ability of specific strains to establish disease in specific populations, the age-specific expression on cells of receptors for toxins, and diameters of renal blood vessels [19].

Analyzed data on outbreaks reported to European Food Safety Authority from 2012 to 2017 showed that 24% of STEC outbreaks are produced from “bovine meat and products thereof” and 22% to “milk and other dairy products.” The third contributor to STEC cases was “tap water, including well water” 13% [6]. Rural areas are more predisposed than urban ones. The global incidence of STEC-associated diseases oscillates extensively, mostly concerning environmental and agricultural factors such as stockbreeding [7].

STEC is a predominant cause of diarrheal illness globally. A metanalysis, with databases from 10 of 14 World Health Organization subregions, estimated a global incidence of 2.8 million cases per year and 3890 HUS cases annually, with a slight decrease in its incidence since 2000 [6]. In the United States, the Centers for Disease Control and Prevention (CDC) reported that the incidence of STEC infection was 5.9 per 100,000 persons during 2018, a 26% increase over the incidence from 2015 to 2017 [20]. This increase reflects the increasing ability to detect non-O157:H7 STEC. New Zealand announced a mean annual incidence of 3.3 per 100,000 persons, whereas Australia only revealed 0.4 cases per 100,000 persons [21].

On the other hand, Latin America has an endemic STEC infection, with most of the cases located in the south of the continent (Argentina, Chile, Uruguay) [22]. It has been estimated that STEC infections in South America cause approximately 2% of acute diarrhea cases and 20-30% of BD [22]. Having had the highest infection rates worldwide, Argentina's incidence during the period 2000-2010 was 7.8-17 per 100,000 children less than five years of age, and the lethality ranged between 2% and 5% (approximately, 10-fold higher than that in other industrialized countries) [22]. The Integrated Bulletin of Statistics of the Ministry of Health of Argentina of February 2019 reported that in 2018 there were 319 cases of HUS [23]. Whereas, between 2000 and 2010, approximately 500 HUS cases were reported annually on average [22]. A total of 260 of those cases (82%) correspond to children under five years of age. This number is lower than the median and average number of cases for the same period of the last eight years, 2010-2017, (375 and 378, respectively). In this age group, 295 cases were reported for a median annual period between 2010 and 2017, with an estimated eight cases per 100,000 cases under five years [23]. The incidence in 2018 was 0.72 cases per 100,000 inhabitants and 6.92 cases per 100,000 under five years [23].

Molecular studies showed that O157 strains from different regions of Argentina present eae, ehxA, and Stx2a/Stx2c as predominant genotypes in both human and bovine samples [24]. The *Stx2a* and *Stx2c* subtypes are frequently associated with BD and HUS cases, but the risk of developing HUS after infection with STEC of the *Stx2a *genotype has been significantly lower than after infection with STEC of the* Stx2c*genotype [19]. These strains, whose predominant *Stx* genotype is *Stx2a + Stx2c*, have acquired factors that increase their ability to cause severe disease and circulate almost exclusively in Argentina, representing more than 80% of human clinical isolates [25]. The high circulation in our country of these hypervirulent strains could partly explain HUS's high incidence in Argentina [22]. More than 80% of human strains showed six of the seven “putative virulence factors” [26]. The q933 allele, which has been related to high toxin production, was present in 98.2% of clinical strains and 75.9% of the bovine isolates [26].

The STEC National Reference Laboratory (“Dr. Carlos G. Malbrán”) analyzes the *E. coli* circulation patterns in Argentina since 1988 by pulsed-field gel electrophoresis (PFGE). AREXHX01.011 pattern was the most common pattern, associated with human disease and food and animals, with circulation throughout the country [23]. This pattern corresponds to *E. coli *O157 strains carrying the *Stx2/ Stx2c* genotype with high pathogenic power. However, from 2015 to August 2018, greater circulation of strains AREXHX01.0650 was observed, displacing the AREXHX01.011 pattern in frequency [23]. The first *E. coli* O157: H7 strain of the AREXHX01.0650 pattern, genotype *Stx2a/Stx2c/eae/ehxA,* was detected in the province of Mendoza in 2002, isolated from a case of diarrhea. Until 2012, the frequency of AREXHX01.0650 detection was one or two cases of human disease per year. However, from 2013 to August 2018, an increase in cases associated with this pattern was observed (seven to 12 cases per year). In the years from 2015 to 2017, strains of *E. coli *O157: H7, pattern AREXHX01.0650, displaced the AREXHX01.011 pattern, mainly in the provinces of the southern region of the country (Neuquén, Chubut and Río Negro, 40% of the total) with more pathogenic and transmissibility capacity [23].

Analysis by either SNPs identified a high predominance of the hypervirulent clade 8, representing more than 80% of clinical isolates in Argentina [25,26]. In contrast, other countries have clade 7 as prevalent [25]. A lineage genetic assay showed a preponderance of lineage I/II, which is linked to the severity of the infections and the high probability to get HUS [26].

Otherwise, STEC non-O157 circulating in Argentina's cattle has shown a high pathogenic virulence. In the last years, there have been notable changes in the whole world's epidemiology, with an increase of incidence in cases of human disease due to STEC non-0157 close to 60% in some serotypes [20]. The serogroup distribution detected in the HUS cases in Argentina corresponds to STEC O157 in 74% of the cases, while non-O157 serogroups cause the remaining 26% [22]. The serogroups non-O157 STEC that are most prevalent in the country are O145, O26, O174, and O121, being the O174 emerging pathogen [22]. In 2012, Carbonari et al. reported that the frequency of non-O157 STEC in Argentina (over 1245 characterized strains) was O121: H19 (2.2%), O145: [H27, NM, NT] (13.6%), O174: [H8, 21, 28, NM] (1.0%), O26: [H2, 11, NT) (1.4%), O103: [H2, NM, NT] (0.6%), O111: [NM, NT] (0.8%), O91: [H21, NM, NT] (0.4%), O8: [H16, 19] (0.4%), ONT: [H6, 7, 11, 12, 49, NT] (3.1%), OR: [H11, NM, NT] (0.6%), and O113: [H4, 19, 21] (0.3%) [27]. In the present study, STEC non-O157 strains were recovered in 28.5% of cases, and the most frequent serotype was O145: NM. In 2016, the National Laboratory (Dr. Carlos G. Malbrán) reported that 70.5% of 396 HUS cases analyzed in the 2011-2015 period corresponded to the O157: H7 serotype and the second most important serotype was O145 (19.4%) [23]. The importance of this serotype, and O26: H11, O103: H2, O111: NM, and O113: H21, lies in its ability to cause severe human disease and epidemic outbreaks [6]. Assay of clinical-relevant cases described the presence of *Stx2a* genotype in an important amount of O26:H11 strains and that O113:H21 strains express mainly *Stx2a *alone or together with *Stx2c* [28]. Furthermore, the O145 serogroup was reported expressing *Stx2a *in bovine and human isolates [28]. Another analysis of pediatric isolates found a higher prevalence of O157:H7, followed by O145:NM in HUS than in diarrheic patients, in Argentina [29]. In addition, O174: H21 isolated from the same study showed that they are negative for eae and positive for STx2a and aggR [29].

Since the Shiga toxin-producing enteroaggregative *E. coli* (Stx-EAEC) O104:H4 strain caused a massive outbreak across Europe in 2011, the importance of the new strain Stx-EAEC has attracted warn attention. A total of 3842 confirmed cases, including 845 HUS cases and 54 deaths, were reported [6]. The epidemic showed that EAEC could become highly virulent through the acquisition of an *Stx2* phage. Last year a case of STx-EAEC was reported in Argentina with the serotype O59:HNM [H19] [30]. In Argentina, this is the first case report of HUS associated with a StxEAEC infection. The strain harbored *Stx2a* associated with STEC and *AggR* genes associated with EAEC [30].

## Conclusions

There are many contributory factors of the pathophysiology of the HUS such as the safety of food, host factors (age, immune status, treatments, medical history), and gene expression levels. However, the relationship between Stx genes, adherence, and other virulence genes or gene combinations can suggest general trends of disease outcome. The high HUS rates, high morbidity, and STEC's virulence features in Argentina have been discussed in this study, which are more important than testing food safety in the prevention of contagion vias. All these factors demand that the application of molecular subtyping techniques in real-time is essential for detecting and controlling outbreaks on time. It has been demonstrated that it is possible to apply molecular subtyping techniques in an HD and HUS, although it is necessary to consolidate the cases' exhaustive epidemiological analysis. Furthermore, early diagnosis of infection helps make medical decisions (correct fluid management, rational use of antibiotics) that can avoid severe consequences in case of progression to HUS. Early diagnosis allows us to avoid secondary transmission and prevent probable outbreaks. Furthermore, these pathology characteristics can be used as targets for a new treatment to research. 

## References

[1] Croxen MA, Law RJ, Scholz R, Keeney KM, Wlodarska M, Finlay BB (2013). Recent advances in understanding enteric pathogenic Escherichia coli. Clin Microbiol Rev.

[2] Sadiq SM, Hazen TH, Rasko DA, Eppinger M (2014). EHEC genomics: past, present, and future. Microbiol Spectr.

[3] Riley LW, Remis RS, Helgerson SD (1983). Hemorrhagic colitis associated with a rare Escherichia coli serotype. N Engl J Med.

[4] Karmali MA, Petric M, Steele BT, Lim C (1983). Sporadic cases of haemolytic-uraemic syndrome associated with faecal cytotoxin and cytotoxin-producing Escherichia coli in stools. Lancet.

[5] Wolf MK (1997). Occurrence, distribution, and associations of O and H serogroups, colonization factor antigens, and toxins of enterotoxigenic Escherichia coli. Clin Microbiol Rev.

[6] Pane EB, Koutsoumanis K, Allende A (2020). Pathogenicity assessment of Shiga toxin-producing Escherichia coli (STEC) and the public health risk posed by contamination of food with STEC. EFSA J.

[7] Majowicz SE, Scallan E, Jones-Bitton A (2014). Global incidence of human Shiga toxin-producing Escherichia coli infections and deaths: a systematic review and knowledge synthesis. Foodborne Pathog Dis.

[8] Franz E, Rotariu O, Lopes BS (2019). Phylogeographic analysis reveals multiple international transmission events have driven the global emergence of Escherichia coli O157:H7. Clin Infect Dis.

[9] Bono JL, Keen JE, Clawson ML, Durso LM, Heaton MP, Laegreid WW (2007). Association of Escherichia coli O157:H7 tir polymorphisms with human infection. BMC Infect Dis.

[10] Manning SD, Motiwala AS, Springman AC (2008). Variation in virulence among clades of Escherichia coli O157:H7 associated with disease outbreaks. Proc Natl Acad Sci USA.

[11] Abu-Ali GS, Ouellette LM, Henderson ST, Lacher DW, Riordan JT, Whittam TS, Manning SD (2010). Increased adherence and expression of virulence genes in a lineage of Escherichia coli O157:H7 commonly associated with human infections. PLoS One.

[12] Kulasekara BR, Jacobs M, Zhou Y (2009). Analysis of the genome of the Escherichia coli O157:H7 2006 spinach-associated outbreak isolate indicates candidate genes that may enhance virulence. Infect Immun.

[13] Lan R, Alles MC, Donohoe K, Martinez MB, Reeves PR (2004). Molecular evolutionary relationships of enteroinvasive. Infect Immun.

[14] Nataro JP, Kaper JB (1998). Diarrheagenic Escherichia coli. Clin Microbiol Rev.

[15] Waldor MK, Friedman DI (2005). Phage regulatory circuits and virulence gene expression. Curr Opin Microbiol.

[16] McGannon CM, Fuller CA, Weiss AA (2010). Different classes of antibiotics differentially influence Shiga toxin production. Antimicrob Agents Chemother.

[17] Lee MS, Tesh VL (2019). Roles of Shiga toxins in immunopathology. Toxins (Basel).

[18] Zhang WL, Köhler B, Oswald E (2002). Genetic diversity of intimin genes of attaching and effacing Escherichia coli strains. J Clin Microbiol.

[19] Naseer U, Løbersli I, Hindrum M, Bruvik T, Brandal LT (2017). Virulence factors of Shiga toxin-producing Escherichia coli and the risk of developing haemolytic uraemic syndrome in Norway, 1992-2013. Eur J Clin Microbiol Infect Dis.

[20] Tack DM, Marder EP, Griffin PM (2019). Preliminary incidence and trends of infections with pathogens transmitted commonly through food - Foodborne Diseases Active Surveillance Network, 10 U.S. Sites, 2015-2018. MMWR Morb Mortal Wkly Rep.

[21] Vally H, Hall G, Dyda A, Raupach J, Knope K, Combs B, Desmarchelier P (2012). Epidemiology of Shiga toxin producing Escherichia coli in Australia, 2000-2010. BMC Public Health.

[22] Torres AG, Amaral MM, Bentancor L, Galli L, Goldstein J, Krüger A, Rojas-Lopez M (2018). Recent advances in Shiga toxin-producing Escherichia coli research in Latin America. Microorganisms.

[23] FERRO N, HERTLEIN C, CASTILLO MC. N° 439 Se 06 (2019). Boletin integerado de vigilancia: number 438. [Argentina integrated bulletin of surveillance: number 438]. https://www.argentina.gob.ar/sites/default/files/biv_438.pdf.

[24] Carbonari CC, Fittipaldi N, Teatero S (2016). Whole-genome sequencing applied to the molecular epidemiology of Shiga toxin-producing Escherichia coli O157:H7 in Argentina. Genome Announc.

[25] Mellor GE, Sim EM, Barlow RS (2012). Phylogenetically related Argentinean and Australian Escherichia coli O157 isolates are distinguished by virulence clades and alternative Shiga toxin 1 and 2 prophages. Appl Environ Microbiol.

[26] Pianciola L, D'Astek BA, Mazzeo M, Chinen I, Masana M, Rivas M (2016). Genetic features of human and bovine Escherichia coli O157:H7 strains isolated in Argentina. Int J Med Microbiol.

[27] Carbonari C, Chinen I, Miliwebsky E (2012). LEE-negative Shiga toxin-producing Escherichia Coli strains associated with human disease in Argentina. Zoonoses Public Health.

[28] Krüger A, Burgán J, Friedrich AW, Rossen JW, Lucchesi PM (2018). ArgO145, a Stx2a prophage of a bovine O145:H- STEC strain, is closely related to phages of virulent human strains. Infect Genet Evol.

[29] Oderiz S, Leotta GA, Galli L (2018). Detection and characterization of Shiga toxin-producing Escherichia coli in children treated at an inter-zonal pediatric hospital in the city of La Plata. [Article in Spanish]. Rev Argent Microbiol.

[30] Carbonari CC, Ricciardi M, Calvo AR (2020). An Stx-EAEC O59:NM[H19] strain isolated from a hemolytic uremic syndrome case in Argentina. Rev Argent Microbiol.

